# Impairment of Quality of Life among Patients with Wearable Cardioverter Defibrillator Therapy (LifeVest®): A Preliminary Study

**DOI:** 10.1155/2018/6028494

**Published:** 2018-06-27

**Authors:** Korbinian Lackermair, Christoph G. Schuhmann, Michaela Kubieniec, Lisa M. Riesinger, Ina Klier, Thomas J. Stocker, Stefan Kääb, Heidi L. Estner, Stephanie Fichtner

**Affiliations:** Department of Cardiology, Medizinische Klinik und Poliklinik I, University Hospital Munich, Ludwig Maximilians University, Germany

## Abstract

**Background:**

Wearable cardioverter defibrillator (WCD) therapy is feasible and safe in patients as a transient protection against sudden cardiac death (SCD). However, the impact of WCD therapy on quality of life (QoL) has not been studied.

**Methods:**

In our single-centre study, 109 consecutive patients with a prescription of WCD were retrospectively analysed. Quality of life has been assessed by a standardized questionnaire (EQ-5D-3L, modified). Additionally, clinical baseline and follow-up data and recorded arrhythmic episodes were evaluated.

**Results:**

Mean WCD therapy time was 56.2 (± 42.4) days, with a daily wear time of 19.7 (± 5) hours. A total of 3441 arrhythmia episodes were detected. Of these, 27 (1%) were adequate but did not require shock therapy. Likewise, no inadequate shock therapy occurred. WCD therapy negatively affected quality of life: 43% of patients reported mental health issues. 37% reported pain or discomfort. Self-care, usual activities, and mobility were restricted in 17%, 48%, and 36%, respectively. 29% were afraid of receiving shock therapy, and 48% suffered from sleep disturbance. However, 64% indicated having felt safe during WCD therapy. Accordingly, average quality of life was rated 70/100 points.

**Conclusion:**

In our cohort, no SCD was prevented by WCD therapy. In contrast, in this preliminary study quality of life was reduced. Thus, careful recommendation of WCD therapy for high risk patients should be considered.

## 1. Background

Sudden cardiac death (SCD) causes approximately 4.25 million deaths every year worldwide [1]. Implantable cardioverter defibrillator is the established therapy for preventing sudden cardiac death due to ventricular arrhythmia. In recent years, the wearable cardioverter defibrillator therapy (WCD, LifeVest) has been introduced as an option for a transient time period in patients at risk for SCD while permanent ICD therapy is not suitable, e.g., due to new onset of cardiomyopathy or infectious issues. Technical aspects and limitations of WCD, as well as potential areas of technical improvement, were described by Agarwal et al. recently [2]. WCD has been shown to be feasible and safe in prevention of SCD without relevant events of inappropriate therapy [3–7].

In the recently presented randomized VEST trial on 2302 postmyocardial infarction patients (American College of Cardiology Annual Scientific Session/ACC 2018, Orlando, FL, March 10, 2018), prescription of WCD was able to reduce the second endpoint all-cause mortality (reduction of relative risk: 35.5%). However, the primary endpoint of SCD and ventricular arrhythmia death was not significantly different [8].

Accordingly, WCD therapy has been recommended by the guidelines of the European Society of Cardiology in 2015 for the management of patients with ventricular arrhythmias and the prevention of SCD in patients with severely reduced systolic left ventricular ejection fraction who are at risk of SCD but do not qualify for an implantable defibrillator (yet), e.g., patients with new onset myocarditis or cardiomyopathy and patients with arrhythmias in the early phase postmyocardial infarction or as a bridge to (a new) transvenous ICD implantation.

For both WCD and ICD therapy, many patients have to be treated with the WCD to prevent one SCD. WCD is an expensive therapy and is accompanied by problems with reimbursement, as previously reported in a European multicentre experience [9].

Moreover, all patients treated with the WCD have to take upon the associated discomfort (e.g., weight of the defibrillator vest, sleep disturbance, and sexual intercourse), while only very few are actually saved from SCD. Although this may obviously have an impact on patients QoL, the effect of WCD therapy on QoL has not been studied methodically yet. Thus, we present our preliminary experience with a cohort of WCD patients with a special focus on the influence on QoL.

## 2. Methods

We conducted a single-centre retrospective investigation of consecutive patients who were prescribed with a WCD (LifeVest 4000) manufactured by Zoll, PA, USA, from March 2012 to February 2016.

WCD therapy was prescribed to patients with high risk for SCD who were not eligible for ICD therapy at the time of diagnosis, at the discretion of the treating cardiologist. Data were collected from routine clinical management of in-hospital patients and also in outpatient settings. Baseline characteristics were raised at the initial presentation before beginning of WCD therapy. Every patient presented at least one more time, most commonly in an outpatient setting, at the end of WCD wear time. At this time point, echocardiographic reevaluation of left ventricular ejection fraction (LVEF), detailed anamnesis, and a physical examination were performed. Conclusively, a decision on ICD implantation was made. Within the scope of anamnesis, QoL was assessed with a standardized questionnaire (EQ-5D-3L, as described elsewhere [10]). The questionnaire was extended by dichotomous questions concerning fear of shock, feeling safe, sleep disturbance, and impairment of usual activities subjectively caused specifically by WCD therapy.

The retrospective analysis was conducted in accordance with the declaration of Helsinki and was approved by the ethics committee of the University of Munich (project number 17-662).

All data are presented as mean ± SD for continuous variables. For categorical variables number of cases or percentage was reported. T tests were performed for comparison of continuous variables. Categorical variables were compared using a chi-squared test. A p value < 0.05 was considered statistically significant. Statistical analyses were performed with SPSS software, version 23, IBM Corp., Armonk, NY, USA.

## 3. Results

A total of 109 consecutive patients were included in our study. Baseline characteristics of our cohort are depicted in Table 1. The predominant indication for WCD prescription was primary prophylactic. Patients had symptomatic systolic heart failure with reduced LVEF. Correspondingly, current medication consists of medical heart failure therapy. 78 patients presented with newly diagnosed nonischemic or ischemic cardiomyopathy or were after acute myocardial infarction. 31 patients already had an ICD implanted but were temporarily not eligible for ICD therapy because of ICD explantation due to device infection or complications with ICD leads.

Mean wear time of the WCD was 56.2 ± 42.4 days, with a daily wear time of 19.7 ± 5.0 hours per day.

In total, 3441 episodes were detected. The vast majority of episodes were inadequate (3414, 99%), mostly due to noise (2734, 80%), oversensing (659, 19%), and supraventricular tachycardia (21, <1%). There were 27 (<1%) adequate episodes due to sustained VTs in four patients (two patients after acute myocardial infarction, one patient with ICM but without acute myocardial infarction, and one patient with myocarditis). No single adequate or inadequate shock therapy occurred, either because episodes terminated spontaneously or therapy was inhibited by the patients by pressing the response button of the WCD.

All patients with preexisting ICD therapy received de novo implantation of an ICD after WCD therapy.

LVEF had improved significantly at the end of WCD wear time (31.8 ± 13.9 versus 37.6 ± 13.1; p<0.01). Accordingly, only 35 of 78 patients without preexisting ICD therapy received de novo ICD implantation.

Complete data on QoL (modified EQ-5D-3L questionnaire, as described above) was accessible in 87 patients (all 78 patients without prior ICD therapy and 9 patients after ICD explantation).

**Figure 1:**
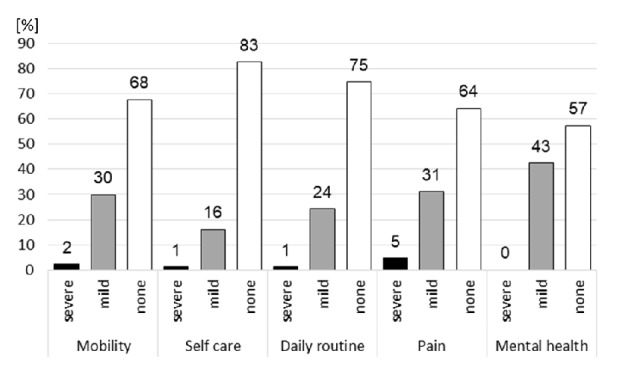
The five standard items of EQ-5D-3L are depicted [percentage of patients]. Severe impairments are shown in black bars and mild impairments in grey bars. Patients stating no impairment are depicted in white bars.

The levels of impairment of the 5 different items of EQ-5D-3L are depicted in Figure 1. Mobility was severely reduced in 2% and mildly in 30%. The ability of self-care (e.g., washing and dressing) was severely diminished in 1% and mildly in 16%. 1% of patients reported having severe problems in accomplishing their daily routine activities (e.g., job, housekeeping), and 24% had mild problems. Severe pain was reported by 5% and mild pain by 31%, respectively. 43% had mild mental health (e.g,. depression and anxiety) issues, whereas no patient reported severe mental health issues.

The overall subjective state of health, on a visual analogue scale from 0 to 100 points, was averagely 70 points.

**Figure 2:**
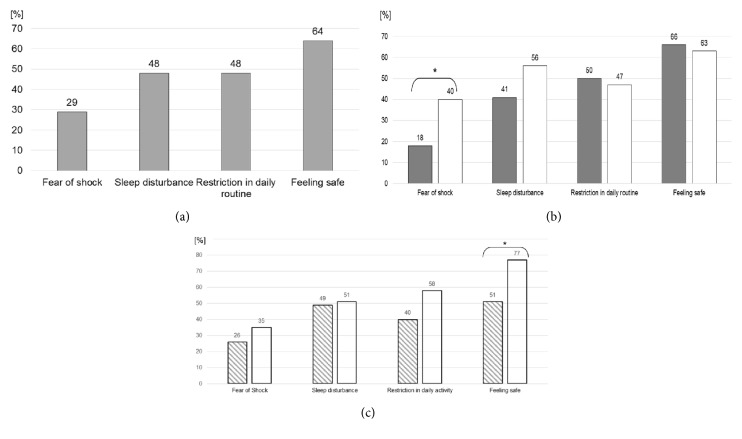
The additional items referring to WCD related QoL are depicted [percentage of patients]. (a) Without subgroups. (b) With subgroup differentiation comparing patients with 3 or less WCD warning signals (grey bar; n=44) to patients with at least 3 WCD warning signals (white bars; n=43). (c) With subgroup differentiation comparing patients with (striped bars; n=35) and without (white bars: n=43) ICD implantation after WCD therapy.

The dichotomous questions specifically addressing the subjective perception during WCD therapy revealed that 29% were afraid of receiving shock therapy. WCD related sleep disturbance or impairment of daily routine activities was reported by 48%. Only 64% reported feeling adequately protected by the WCD (Figure 2(a)).

Additionally, the influence of the number of warning signals that occurred during WCD therapy on the dichotomous items was analysed. 44 patients experienced 3 (mean 0.7±1) or less warning signals, and 43 experienced more than 3 (mean 72±147), respectively. More warning signals significantly increased fear of shock therapy (18 versus 40%; p=0.03), and there was a trend towards increased sleep disturbance (41 versus 56%; p=0.16). Subjective impairment of daily routine activities (50 versus 47%; p=0.75) and the feeling of being protected (66 versus 63%; p=0.76) were not affected by the number of warning signals, at all (Figure 2(b)).

35 of 78 patients without prior ICD therapy received an ICD after the WCD therapy. Compared to patients with ICD implantation, patients without ICD implantation at the end of WCD therapy reported having felt more safe (77 versus 51%; p<0.01) without significant differences of the fear of shock (35 versus 26%; p=0.39), WCD related sleep disturbance (51 versus 49%; p=0.8), and restriction of daily activities (58 versus 40%; p=0.11; Figure 2(c)).

## 4. Discussion

To the best of our knowledge, this is the first study to examine the impact of WCD therapy on QoL. As Sears et al. stated, although no universal definition of QoL exists, QoL is a generic term for a multidimensional health outcome, in which biological, psychological, and social functioning are interdependent [11]. In summary, our major findings are that in a standardized QoL questionnaire every 3^rd^ to 6^th^ patient reported mild issues and 1 to 2 percent complained about severe issues. Of note, in dichotomous questions specifically addressing the subjective situation during WCD use, one-third of patients reported fear of shock treatment and half of patients felt disturbed during their sleep and restricted in daily routine activities. Furthermore, there was a correlation between number of alarms given by the WCD for pending shock therapy and patients who reported QoL issues. This stands in contrast to a short report by the manufacturer of the WCD LifeVest (Zoll) [12]. In the survey, patients reported by telephone interview to predominantly feel well protected (88%), 82% felt more confident in daily routine activities, and 72% reported an improvement in sleep, whereas our study shows a substantial impairment of QoL associated with all these features. As the most alarms were given for inadequate episodes, it is obvious that development is needed to decrease the number of inadequate episodes and inadequate therapy warning signals to decrease the patients fear during WCD therapy.

Interestingly, we observed a discrepancy in the perception of QoL when patients were asked about their general condition and their condition with specific view on the situation under WCD therapy. For example, when patients were asked to rate their restrictions in daily routine, the same patients at the same time considered their restrictions more severe when specifically asked about WCD therapy (Figures 1 and 2(a)).

In our cohort, 78 patients received a WCD without existing prior ICD therapy. Taken into consideration that only 35 patients required ICD implantation at the end of WCD therapy (meaning that the remaining 43 patients who did not receive an ICD presumably suffered from less severe heart failure at the end of WCD therapy), impairment of QoL might have been predominantly caused by WCD therapy. Interestingly, patients without ICD implantation more often reported having felt safe during WCD therapy. This might be confounded by the patient's relief after an improvement of systolic function. Nevertheless, impairment of the other items (sleep disturbance, restriction of daily activity, and fear of shock) was even more pronounced in these patients.

Comparing our findings with data on QoL in patients with ICD therapy, we found that the results of randomized trials are sparse and difficult to compare as no standard tool for measurement of QoL exists [11, 13–16]. To sum up, QoL in ICD patients who do not receive shock therapy resembles that in non-ICD patients, while QoL in ICD patients who experience shock therapy is diminished [16]. Again, impairment of QoL may be driven by shock therapy and not the presence of heart failure itself. Comparing patients with ICD to patients without ICD but with antiarrhythmic drug therapy, it has been shown that QoL is maintained in ICD treated patients but diminished in patients with antiarrhythmic therapy. This states the impression that ICD therapy (without shock therapy) is beneficial for QoL.

In contrast our findings are suggestive of the idea that WCD therapy without shock therapy falls short of these beneficial effects on QoL resulting in poor compliance with an average daily wear time of only 82%. Our findings are in accordance with the latest presented results from the VEST trial. In this trial 2302 patients after acute myocardial infarction and an impairment of left ventricular ejection fraction <35% were randomized to standard care plus WCD or standard care (in a 2:1 ratio). Mean wear time of all for WCD therapy randomized patients was only 59%. The authors hypothesized that this could be due to side effects like rash (12.9 of WCD patients versus 3.8% of control patients; p<0.001) or itching on torso (14.5 of WCD patients versus 3.1% of control patients; p<0.001) [8].

As we know from other studies on WCD therapy [3–5, 17, 18], the overall incidence rate of shock therapy is low; therefore the number needed to treat to save one patient from SCD by WCD therapy will be quite high and it is an expensive therapy (in Germany the monthly cost amounts to roughly 2650 euros). In addition, WCD therapy remarkably diminishes QoL in our collective. Therefore proper patient selection is crucial. A WCD should not be administered to every patient with severely reduced LVEF. Instead, which patients are most suitable for and profit most from WCD therapy has to be determined having regard to current trials like, for example, the Danish trial [19], and results of upcoming prospective trials in patients with de novo diagnosis of severe cardiomyopathy who are at high risk for arrhythmic SCD.

### 4.1. Limitations

Our study has several important limitations. First the study design was retrospective and our cohort is very heterogeneous. That means not all patients were prescribed with a WCD for new onset of severe systolic heart failure for a certain time period, but there were also subjects in our cohort who already had permanent ICD therapy and were equipped with the WCD for a short time to bridge to surgery, e.g., for ICD lead complications. Second, QoL data could not be acquired from every subject. Therefore, it cannot be excluded that there is a certain reporting bias. In addition, complete QoL questionnaire was only available in nine patients with explanted ICD.

Third, our analysis is lacking in a control group of patients with ICD or a control group with heart failure but without ICD or WCD. Therefor we cannot rule out an impairment of QoL due to the heart failure itself.

## 5. Conclusion

In our cohort, no SCD was prevented by WCD therapy. In contrast, in this preliminary study quality of life was reduced. Thus, careful recommendation of WCD therapy for high risk patients should be considered and strategies should be developed to decrease patients' discomfort and increase patients compliance by optimising the WCD fit, specific patient information, and therapy of anxiety in patients at high risk for SCD. Besides, technical development of WCD is needed to decrease the number of inadequate episodes.

## Figures and Tables

**Table 1:** Patient's characteristics.

	**All patients**	**ICM**	**DCM**	**Others**
n	109	46 (42%)	47 (43%)	16 (15%)
Age (years)	58 ± 16	68 ± 16	54 ± 16	44 ± 16
Male sex (%)	85 (78%)	42 (91%)	34 (72%)	9 (56%)
After acute myocardial infarction	16 (15%)	16 (35%)	0	0
LV-EF (%)	32 ± 14	36 ± 15	26 ± 9	38 ± 17
Indication				
Primary prophylactic	81 (74%)	29 (63%)	42 (89%)	10 (63%)
Secondary prophylactic	38 (26%)	17 (37%)	5 (11%)	6 (37%)
Functional status				
NYHA I	11 (10%)	7 (15%)	1 (2%)	3 (19%)
NYHA II	44 (40%)	23 (50%)	15 (32%)	5 (31%)
NYHA III	44 (40%)	16 (35%)	22 (47%)	6 (37%)
NYHA IV	9 (8%)	0 (0%)	8 (17%)	1 (6%)
Cardiovascular risk factors				
Diabetes mellitus	30 (28%)	17 (37%)	11 (23%)	2 (13%)
Smoking	48 (44%)	24 (52%)	18 (38%)	6 (38%)
Hypercholesterolaemia	52 (48%)	32 (70%)	16 (34%)	4 (25%)
Hypertension	55 (50%)	34 (73%)	17 (36%)	4 (25%)
Creatinine (mg/dl)	1.2 ± 0.6	1.2 ± 0.6	1.3 ± 0.6	1.0 ± 0.6
Current medication				
Beta blocker	103 (94%)	45 (98%)	45 (96%)	13 (81%)
Inhibitors of ACE or AR	95 (87%)	42 (91%)	42 (89%)	11 (69%)
Diuretics	86 (79%)	32 (70%)	45 (96%)	9 (57%)
Aldosterone antagonist	85 (78%)	34 (74%)	41 (87%)	10 (63%)
Amiodarone	7 (6%)	2 (4%)	5 (11%)	0 (0%)

NYHA: New York heart association, ACE: angiotensin converting enzyme, AR: angiotensin receptor.

## Data Availability

The data used to support the findings of this study are included within the article.

## Abbreviations

DCM: Dilated cardiomyopathy
ICM: Ischemic cardiomyopathy
LVEF: Left ventricular ejection fraction
QoL: Quality of life
SCD: Sudden cardiac death
VT: Ventricular tachycardia
WCD: Wearable cardioverter defibrillator.
